# 5-(2-Fluoro­benzyl­idene)-2,2-dimethyl-1,3-dioxane-4,6-dione

**DOI:** 10.1107/S1600536809038811

**Published:** 2009-09-30

**Authors:** Wu-Lan Zeng, Fang-Fang Jian

**Affiliations:** a MicroScale Science Institute,Department of Chemistry and Chemical Engineering, Weifang University, Weifang 261061, People’s Republic of China; b MicroScale Science Institute, Weifang University, Weifang 261061, People’s Republic of China

## Abstract

The title compound, C_13_H_11_FO_4_, was prepared by the reaction of 2,2-dimethyl-1,3-dioxane-4,6-dione and 2-fluoro­benzaldehyde in ethanol. In the crystal structure, mol­ecules are linked into chains by weak inter­molecular C—H⋯O hydrogen bonds.

## Related literature

For background to the use of Meldrum’s acid as a reagent in organic synthesis, see: Kuhn *et al.* (2003 ▶); Casadesus *et al.* (2006 ▶).
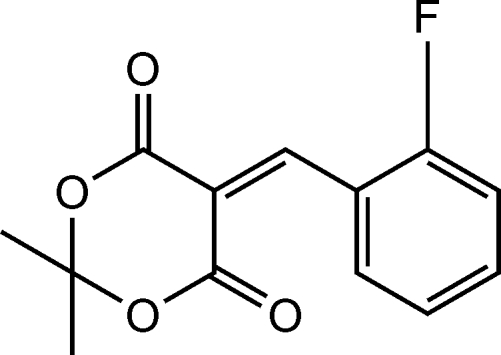


## Experimental

### 

#### Crystal data

C_13_H_11_FO_4_
*M* *_r_* = 250.22
Triclinic, 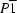

*a* = 5.9907 (12) Å
*b* = 7.6135 (15) Å
*c* = 13.712 (3) Å
α = 104.66 (3)°
β = 97.00 (3)°
γ = 98.50 (3)°
*V* = 590.0 (2) Å^3^
*Z* = 2
Mo *K*α radiation
μ = 0.12 mm^−1^
*T* = 293 K
0.18 × 0.15 × 0.10 mm

#### Data collection

Bruker SMART CCD diffractometer
Absorption correction: none
5846 measured reflections
2680 independent reflections
2232 reflections with *I* > 2σ(*I*)
*R*_int_ = 0.015

#### Refinement

*R*[*F* ^2^ > 2σ(*F* ^2^)] = 0.040
*wR*(*F* ^2^) = 0.122
*S* = 1.09
2680 reflections
163 parameters
H-atom parameters constrained
Δρ_max_ = 0.29 e Å^−3^
Δρ_min_ = −0.21 e Å^−3^

### 

Data collection: *SMART* (Bruker, 1997 ▶); cell refinement: *SAINT* (Bruker, 1997 ▶); data reduction: *SAINT*; program(s) used to solve structure: *SHELXS97* (Sheldrick, 2008 ▶); program(s) used to refine structure: *SHELXL97* (Sheldrick, 2008 ▶); molecular graphics: *SHELXTL* (Sheldrick, 2008 ▶); software used to prepare material for publication: *SHELXTL*.

## Supplementary Material

Crystal structure: contains datablocks global, I. DOI: 10.1107/S1600536809038811/lh2910sup1.cif

Structure factors: contains datablocks I. DOI: 10.1107/S1600536809038811/lh2910Isup2.hkl

Additional supplementary materials:  crystallographic information; 3D view; checkCIF report

## Figures and Tables

**Table 1:** Hydrogen-bond geometry (Å, °)

*D*—H⋯*A*	*D*—H	H⋯*A*	*D*⋯*A*	*D*—H⋯*A*
C1—H1*A*⋯O1^i^	0.93	2.42	3.343 (4)	174

Symmetry code: (i) 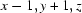.

**Fig. 1.**
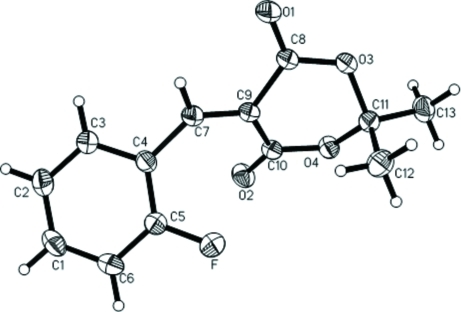
The molecular structure of (I), drawn with 30% probability ellipsoids and spheres of arbritrary size for the H atoms.

## supplementary crystallographic information

### Comment

Starting with its discovery and correct structural assignment, Meldrum's acid has become a widely used reagent in organic synthesis (Kuhn *et al.*, 2003; Casadesus *et al.*, 2006) owing to the interesting conformational features of the products. We report here the synthesis and structure of the title compound,(I) (Fig. 1). The crystal structure analysis confirms the structure of the title compound with atom C7 connected to the 1,3-dioxane ring via a C4-C7 single bond [1.464 (2)Å] and the phenyl ring via a C7═C9 double bond [1.334 (2)Å]. The crystal structure is stabilized by weak intermolecular C—H···O hydrogen bonds (Table 1).

### Experimental

The mixture of malonic acid (6.24 g, 0.06 mol) and acetic anhydride(9 ml) in strong sulfuric acid (0.25 ml) was stirred with water at 303K, After dissolving, propan-2-one (3.48 g, 0.06 mol) was added dropwise into solution for 1 h. The reaction was allowed to proceed for 2 h. The mixture was cooled and filtered, and then an ethanol solution of 2-fluorobenzaldehyde (7.67g,0.06 mol) was added. The solution was then filtered and concentrated. Single crystals were obtained by evaporation of an petroleum ether-ethylacetate (2:1 *v*/*v*) solution of (I) at room temperature over a period of one week.

### Refinement

The H atoms were placed in calculated positions (C—H = 0.93–0.96 Å), and refined as riding with *U*_iso_(H) = 1.2*U*_eq_(C) or 1.5U_eq_(methyl C).

### Crystal data

**Table:**

C_13_H_11_FO_4_	*Z* = 2
*M**_r_* = 250.22	*F*(000) = 260
Triclinic, *P*1	*D*_x_ = 1.408 Mg m^−^^3^
Hall symbol: -P 1	Mo *K*α radiation, λ = 0.71073 Å
*a* = 5.9907 (12) Å	Cell parameters from 2680 reflections
*b* = 7.6135 (15) Å	θ = 3.1–27.5°
*c* = 13.712 (3) Å	µ = 0.12 mm^−^^1^
α = 104.66 (3)°	*T* = 293 K
β = 97.00 (3)°	Block, colorless
γ = 98.50 (3)°	0.18 × 0.15 × 0.10 mm
*V* = 590.0 (2) Å^3^	

### Data collection

**Table:**

Bruker SMART CCD diffractometer	2232 reflections with *I* > 2σ(*I*)
Radiation source: fine-focus sealed tube	*R*_int_ = 0.015
graphite	θ_max_ = 27.5°, θ_min_ = 3.1°
φ and ω scans	*h* = −7→7
5846 measured reflections	*k* = −9→9
2680 independent reflections	*l* = −17→17

### Refinement

**Table:**

Refinement on *F*^2^	Primary atom site location: structure-invariant direct methods
Least-squares matrix: full	Secondary atom site location: difference Fourier map
*R*[*F*^2^ > 2σ(*F*^2^)] = 0.040	Hydrogen site location: inferred from neighbouring sites
*wR*(*F*^2^) = 0.122	H-atom parameters constrained
*S* = 1.09	*w* = 1/[σ^2^(*F*_o_^2^) + (0.0689*P*)^2^ + 0.0762*P*] where *P* = (*F*_o_^2^ + 2*F*_c_^2^)/3
2680 reflections	(Δ/σ)_max_ < 0.001
163 parameters	Δρ_max_ = 0.28 e Å^−^^3^
0 restraints	Δρ_min_ = −0.21 e Å^−^^3^

### Special details

**Table:**

Geometry. All e.s.d.'s (except the e.s.d. in the dihedral angle between two l.s. planes) are estimated using the full covariance matrix. The cell e.s.d.'s are taken into account individually in the estimation of e.s.d.'s in distances, angles and torsion angles; correlations between e.s.d.'s in cell parameters are only used when they are defined by crystal symmetry. An approximate (isotropic) treatment of cell e.s.d.'s is used for estimating e.s.d.'s involving l.s. planes.
Refinement. Refinement of *F*^2^ against ALL reflections. The weighted *R*-factor *wR* and goodness of fit *S* are based on *F*^2^, conventional *R*-factors *R* are based on *F*, with *F* set to zero for negative *F*^2^. The threshold expression of *F*^2^ > σ(*F*^2^) is used only for calculating *R*-factors(gt) *etc*. and is not relevant to the choice of reflections for refinement. *R*-factors based on *F*^2^ are statistically about twice as large as those based on *F*, and *R*- factors based on ALL data will be even larger.

### Fractional atomic coordinates and isotropic or equivalent isotropic displacement parameters (Å^2^)

**Table:**

	*x*	*y*	*z*	*U*_iso_*/*U*_eq_	
O4	0.24998 (15)	0.52398 (12)	0.08949 (6)	0.0433 (2)	
F	−0.21328 (14)	0.74829 (12)	0.22191 (7)	0.0610 (3)	
O3	0.42130 (16)	0.39182 (13)	0.21066 (7)	0.0487 (2)	
O2	0.19470 (18)	0.81052 (13)	0.13437 (7)	0.0532 (3)	
C10	0.22839 (19)	0.67636 (17)	0.16038 (9)	0.0387 (3)	
O1	0.5140 (2)	0.54637 (16)	0.37345 (8)	0.0694 (4)	
C4	0.0848 (2)	0.93620 (17)	0.35028 (9)	0.0418 (3)	
C11	0.2576 (2)	0.35465 (17)	0.11792 (9)	0.0413 (3)	
C7	0.2194 (2)	0.78997 (18)	0.34775 (9)	0.0436 (3)	
H7A	0.2780	0.7790	0.4115	0.052*	
C9	0.2713 (2)	0.66953 (16)	0.26804 (9)	0.0390 (3)	
C6	−0.2546 (2)	1.0510 (2)	0.29876 (11)	0.0521 (3)	
H6A	−0.3967	1.0306	0.2578	0.063*	
C5	−0.1258 (2)	0.91420 (18)	0.29004 (9)	0.0430 (3)	
C8	0.4099 (2)	0.53311 (18)	0.29079 (10)	0.0455 (3)	
C3	0.1648 (3)	1.1072 (2)	0.42261 (11)	0.0547 (4)	
H3A	0.3034	1.1261	0.4660	0.066*	
C13	0.3522 (3)	0.2316 (2)	0.03544 (11)	0.0592 (4)	
H13A	0.5007	0.2923	0.0301	0.089*	
H13B	0.2516	0.2058	−0.0286	0.089*	
H13C	0.3649	0.1179	0.0520	0.089*	
C2	0.0417 (3)	1.2483 (2)	0.43080 (12)	0.0613 (4)	
H2A	0.1004	1.3627	0.4775	0.074*	
C1	−0.1683 (3)	1.2195 (2)	0.36964 (12)	0.0582 (4)	
H1A	−0.2522	1.3141	0.3762	0.070*	
C12	0.0242 (3)	0.2714 (2)	0.13214 (12)	0.0566 (4)	
H12A	−0.0266	0.3564	0.1856	0.085*	
H12B	0.0317	0.1583	0.1503	0.085*	
H12C	−0.0815	0.2462	0.0696	0.085*	

### Atomic displacement parameters (Å^2^)

**Table:**

	*U*^11^	*U*^22^	*U*^33^	*U*^12^	*U*^13^	*U*^23^
O4	0.0517 (5)	0.0425 (5)	0.0385 (4)	0.0132 (4)	0.0100 (4)	0.0124 (4)
F	0.0480 (5)	0.0557 (5)	0.0669 (5)	0.0051 (4)	−0.0003 (4)	0.0021 (4)
O3	0.0514 (5)	0.0451 (5)	0.0475 (5)	0.0221 (4)	−0.0027 (4)	0.0065 (4)
O2	0.0690 (6)	0.0492 (5)	0.0510 (5)	0.0235 (5)	0.0113 (4)	0.0233 (4)
C10	0.0364 (5)	0.0409 (6)	0.0410 (6)	0.0114 (5)	0.0064 (5)	0.0129 (5)
O1	0.0878 (8)	0.0677 (7)	0.0492 (6)	0.0420 (6)	−0.0159 (5)	0.0068 (5)
C4	0.0452 (6)	0.0436 (7)	0.0389 (6)	0.0171 (5)	0.0077 (5)	0.0103 (5)
C11	0.0442 (6)	0.0387 (6)	0.0402 (6)	0.0099 (5)	0.0034 (5)	0.0098 (5)
C7	0.0463 (6)	0.0461 (7)	0.0387 (6)	0.0170 (5)	0.0013 (5)	0.0100 (5)
C9	0.0390 (6)	0.0393 (6)	0.0394 (6)	0.0128 (5)	0.0017 (5)	0.0114 (5)
C6	0.0409 (6)	0.0620 (9)	0.0593 (8)	0.0191 (6)	0.0085 (6)	0.0218 (7)
C5	0.0411 (6)	0.0440 (7)	0.0440 (6)	0.0089 (5)	0.0080 (5)	0.0110 (5)
C8	0.0485 (7)	0.0431 (7)	0.0441 (6)	0.0183 (5)	−0.0014 (5)	0.0092 (5)
C3	0.0550 (8)	0.0542 (8)	0.0479 (7)	0.0200 (6)	−0.0012 (6)	0.0007 (6)
C13	0.0701 (9)	0.0534 (8)	0.0528 (8)	0.0230 (7)	0.0137 (7)	0.0042 (6)
C2	0.0728 (10)	0.0465 (8)	0.0605 (8)	0.0221 (7)	0.0086 (7)	0.0022 (6)
C1	0.0653 (9)	0.0537 (8)	0.0661 (9)	0.0319 (7)	0.0188 (7)	0.0200 (7)
C12	0.0525 (8)	0.0538 (8)	0.0599 (8)	−0.0008 (6)	0.0093 (6)	0.0155 (6)

### Geometric parameters (Å, °)

**Table:**

O4—C10	1.3460 (15)	C9—C8	1.4904 (17)
O4—C11	1.4434 (15)	C6—C5	1.3753 (19)
F—C5	1.3529 (16)	C6—C1	1.381 (2)
O3—C8	1.3457 (16)	C6—H6A	0.9300
O3—C11	1.4460 (15)	C3—C2	1.380 (2)
O2—C10	1.1996 (15)	C3—H3A	0.9300
C10—C9	1.4826 (16)	C13—H13A	0.9600
O1—C8	1.1979 (16)	C13—H13B	0.9600
C4—C5	1.3843 (18)	C13—H13C	0.9600
C4—C3	1.3978 (19)	C2—C1	1.379 (2)
C4—C7	1.4639 (17)	C2—H2A	0.9300
C11—C13	1.5018 (18)	C1—H1A	0.9300
C11—C12	1.5041 (19)	C12—H12A	0.9600
C7—C9	1.3385 (17)	C12—H12B	0.9600
C7—H7A	0.9300	C12—H12C	0.9600
			
C10—O4—C11	119.32 (9)	C6—C5—C4	123.07 (13)
C8—O3—C11	118.75 (9)	O1—C8—O3	119.85 (12)
O2—C10—O4	119.30 (11)	O1—C8—C9	124.13 (12)
O2—C10—C9	124.62 (11)	O3—C8—C9	115.98 (11)
O4—C10—C9	115.80 (10)	C2—C3—C4	121.18 (14)
C5—C4—C3	116.79 (12)	C2—C3—H3A	119.4
C5—C4—C7	124.51 (12)	C4—C3—H3A	119.4
C3—C4—C7	118.56 (12)	C11—C13—H13A	109.5
O4—C11—O3	109.41 (10)	C11—C13—H13B	109.5
O4—C11—C13	106.34 (11)	H13A—C13—H13B	109.5
O3—C11—C13	106.18 (11)	C11—C13—H13C	109.5
O4—C11—C12	110.47 (11)	H13A—C13—H13C	109.5
O3—C11—C12	110.71 (11)	H13B—C13—H13C	109.5
C13—C11—C12	113.52 (12)	C1—C2—C3	119.93 (14)
C9—C7—C4	130.10 (11)	C1—C2—H2A	120.0
C9—C7—H7A	114.9	C3—C2—H2A	120.0
C4—C7—H7A	114.9	C2—C1—C6	120.39 (13)
C7—C9—C10	125.14 (11)	C2—C1—H1A	119.8
C7—C9—C8	117.26 (11)	C6—C1—H1A	119.8
C10—C9—C8	117.09 (10)	C11—C12—H12A	109.5
C5—C6—C1	118.59 (13)	C11—C12—H12B	109.5
C5—C6—H6A	120.7	H12A—C12—H12B	109.5
C1—C6—H6A	120.7	C11—C12—H12C	109.5
F—C5—C6	118.32 (12)	H12A—C12—H12C	109.5
F—C5—C4	118.57 (12)	H12B—C12—H12C	109.5

### Hydrogen-bond geometry (Å, °)

**Table:**

*D*—H···*A*	*D*—H	H···*A*	*D*···*A*	*D*—H···*A*
C1—H1A···O1^i^	0.93	2.42	3.343 (4)	174
C7—H7A···O1	0.93	2.42	2.798 (2)	104

Symmetry codes: (i) *x*−1, *y*+1, *z*.
